# Deciphering the role of the Sch9 serine/threonine kinase in *Scedosporium apiospermum*

**DOI:** 10.3389/ffunb.2026.1820509

**Published:** 2026-05-11

**Authors:** Samar Kabbara, Hajar Yaakoub, Baptiste Bidon, Charlotte Godon, Bienvenue Razafimandimby, Yves Delneste, Pascale Pignon, Jean-Philippe Bouchara, Monzer Hamze, Nicolas Papon

**Affiliations:** 1 Univ Angers, Univ Brest, IRF, SFR ICAT, Angers, France; 2 Univ Angers, Nantes Université, Inserm, CNRS, CRCI2NA, SFR ICAT, Angers, France; 3 Laboratoire Microbiologie Santé et Environnement (LMSE), Ecole Doctorale des Sciences et de Technologie, Faculté de Santé Publique, Université Libanaise, Tripoli, Lebanon

**Keywords:** functional characterization, fungal fitness, *S. apiospermum*, Sch9, TOR pathway

## Abstract

*Scedosporium apiospermum* is an emerging opportunistic fungal pathogen, primarily affecting immunocompromised individuals. Notably, it ranks second among filamentous fungi infecting cystic fibrosis (CF) patients. Infections caused by *S. apiospermum* can range from localized infections to pulmonary colonization and invasive disseminated infections, often resulting in high mortality rates. This is largely attributed to the patient’s clinical condition, limited diagnostic tools, and resistance to current antifungal therapies. Therefore, ongoing research is crucial to better understand the pathophysiology of these deep-seated fungal infections and to develop new strategies for early diagnosis and more effective antifungal treatments. In the present study, the *Δsch9* gene, which encodes a major effector of the TOR pathway in fungi, was characterized. The deletion of *sch9* significantly impaired *S. apiospermum* fitness under standard growth conditions and reduced hyphal development. Additionally, the *Δsch9* mutant strain displayed increased sensitivity to oxidative stress and cell wall stress-inducing agents. The mutant strain showed an increase in germ tube formation during the early hours of growth, particularly under hypoxic and hypercapnic conditions. Furthermore, under standard growth conditions, the *Δsch9* mutant strain exhibited a decrease in germ tube cell wall thickness compared to the *Δku70* parent strain. Finally, disruption of *Δsch9* affected the *S. apiospermum* ability to resist ingestion and killing by macrophages. Taken together, these findings suggest that Sch9 plays a crucial role in stress tolerance, morphogenesis, and host-pathogen interactions in *S. apiospermum*. Further studies are needed to explore Sch9 as a potential therapeutic target in the treatment of *S. apiospermum* infections.

## Introduction

Cystic fibrosis (CF) is the most common hereditary genetic disease among the Caucasian population in Europe and the third leading rare disease in France. It is caused by mutations in the gene encoding the cystic fibrosis transmembrane conductance regulator (CFTR), a membrane transporter responsible for electrolyte exchange across the plasma membrane of various epithelial cell types. While multiple organs are impacted by the dysfunction of CFTR, the prognosis primarily depends on the extent of respiratory damage. Mutations in the *CFTR* gene result in thickening of the bronchial mucus and alterations in mucociliary clearance, which allows the entrapment of inhaled bacterial and fungal spores and creates an environment favorable to the microbial growth. The resulting infections are the leading cause of morbidity and mortality in CF (Santos-Fernandez et al., 2023). Bacteria, in particular *Pseudomonas aeruginosa*, are the prominent agents of these infections, justifying the specific attention paid to bacterial infections in recent decades. As a result, significant advancement has been made in therapeutic management and prevention of these infections, which, together with the implementation of early diagnosis of CF and the improvement of the nutritional status of patients, has led to a significant increase in life expectancy of patients (Burgel et al., 2024).

In addition to bacteria, various fungal species can colonize the respiratory tract of CF patients. This colonization is facilitated by repeated use of antibiotics and corticosteroids, contributing to the exacerbation of inflammatory responses and potentially leading to actual infections (Saiman, 2004). However, unlike bacterial infections, much less is known about the epidemiology and pathophysiology of fungal infections in CF patients. *Aspergillus fumigatus* is the most common filamentous fungus colonizing the lungs of CF patients. Other fungal species are also increasingly reported in this context, with *Scedosporium apiospermum* ranking second in prevalence (Bouchara and Papon, 2019). Despite this, the pathogenesis of *Scedosporium* infections remains poorly understood, particularly the mechanisms that allow these molds to establish themselves within the bronchial tree. in CF lungs, thick mucus secretions and chronic inflammation may culminate in potential hypoxic and hypercapnic conditions (Bradley et al., 1999), along with prominent oxidative stress (Moliteo et al., 2022), thereby creating a hostile microenvironment. This raises the question of how *S. apiospermum* is able to adapt to such harsh conditions and successfully colonize the respiratory tracts (Bouchara et al., 2020). Part of this question has been addressed in the setting of oxidative stress, with work from our laboratory identifying key components of the antioxidant machinery and key regulators in *S. apiospermum*, including the Hog1 and Skn7 signaling pathways (Yaakoub et al., 2025; Yaakoub et al., 2023; Staerck et al., 2019).

The Target of Rapamycin (TOR) signaling pathway, conserved across all eukaryotes, has emerged as a crucial regulator of fungal growth and metabolism in response to various environmental and nutritional cues (Wang et al., 2023). At the core of this pathway lies the kinase Tor, which functions within multi-protein complexes, namely TOR complex 1 (TORC1) and TORC2 (Wullschleger et al., 2006; Foltman and Sanchez-Diaz, 2023). One of the major substrates of TORC1 is *Sch9* (Scott Cameron HindIII library clone N°9), a serine/threonine kinase belonging to the AGC kinase family often localized in the cytosol and on vacuolar membranes (Sobko, 2006; Caligaris et al., 2023). TORC1-dependent phosphorylation of Sch9 at its C-terminal end results in regulation of growth processes related to nutrient and environmental adaptations, including stress response, ribosome biogenesis, autophagy, and longevity, while nutrient starvation or rapamycin inactivates its activity (Caligaris et al., 2023; Fujii et al., 2025). Studies in yeasts deciphered conserved TORC1-independent role of Sch9 in CO_2_ sensing, involving the regulation of expression of carbonic anhydrase *nce103* via Pkh1/2-Sch9-Cst6/Rca1 signaling cascade (Martin et al., 2017; Pohlers et al., 2017). Still, TORC1-Sch9 is required to repress the hyperfilamentation phenotype in *C. albicans* exposed to hypoxia (<10%) and high CO_2_ levels (>1%) (Stichternoth et al., 2011), which mimic conditions encountered in specific host niches. This regulation may contribute to immune evasion by limiting exposure of the immunogenic hyphal form. Additional studies in *C. albicans* further support a role for the Sch9-Rca1-Nce103 signaling module in pathogen-associated molecular pattern (PAMP) masking in response to these gaseous conditions, as well as contribution of CaSch9 to virulence in mouse models (Stichternoth et al., 2011; Avelar et al., 2024). The involvement of Sch9 in virulence has also been reported in other fungal pathogens, including *Cryptococcus neoformans* (Wang et al., 2004), *Aspergillus fumigatus* (Alves de Castro et al., 2016), and the phytopathogenic *A. flavus* and *Fusarium graminearum* (Gu et al., 2015; Li et al., 2024), highlighting a potentially conserved role of Sch9 in fungal pathogenicity. Otherwise Sch9 has been implicated in a wide range of cellular processes that are conserved, to varying degrees, across fungal species. These include adaptation to osmotic, potentially involving functional interplay with the HOG pathway (Pascual-Ahuir and Proft, 2007; Alves de Castro et al., 2016; Li et al., 2024), as well as responses to oxidative and cell wall stress (Gu et al., 2015), thermotolerance (Yang et al., 2017; Batool et al., 2022), mycotoxin production (Gu et al., 2015; Li et al., 2024), and the regulation of vegetative differentiation (Gu et al., 2015); Kim et al;, 2019; Batool et al., 2022).

Given the central role played by Sch9 in stress adaptation and pathogenicity in many fungal models, we aimed in this study to investigate the role of corresponding gene in *Scedosporium apiospermum*, with a focus on the various stress conditions characteristic of CF lung microenvironment.

## Materials and methods

### Sequence analysis

Sequence analysis were performed following sequence similarity search by Blast, and domains were predicted using the SMART algorithm.

### Strains and standard growth conditions

The wild-type strain of *Scedosporium apiospermum* used in this study is IHEM 14462, whose genome was sequenced in 2014 (Vandeputte et al., 2014). This strain was originally isolated in 1998 from the sputum of a CF patient at the University Hospital of Tours. However, the disruption of *sch9* was performed in the *Δku70* parent strain, which was recently generated from *S. apiospermum* IHEM 14462 to enhance homologous recombination events during the transgenesis protocol. All *S. apiospermum* strains were routinely cultured on yeast peptone dextrose agar (YPDA: yeast extract, 5 g/L; pancreatic peptone, 10 g/L; glucose, 20 g/L; and agar, 20 g/L) or potato dextrose agar (PDA: dextrose, 20 g/L; potato infusion, 200 g/L; and bacteriological agar, 15 g/L) supplemented with 0.5 g/L chloramphenicol. Strains were incubated at 37 °C for 7 to 14 days depending on the assay. Conidia used for subsequent experiments were harvested from 9-day-old cultures. Certain cultures were grown under defined gaseous conditions. To achieve this, a Sci-tive chamber workstation from Baker Ruskinn was used to simulate hypoxia (0.1% CO_2_, 2% O_2_) or hypercapnia (7% CO_2_, 18% O_2_). These gas concentrations fall within the ranges previously reported in the literature to mimic conditions encountered in CF lungs (Peters-Hall et al., 2017; Wong et al., 2023).

### Extraction of genomic DNA

Genomic DNA was extracted from 9-day-old cultures using the standard phenol-chloroform extraction method (Sambrook et al., 1989). The DNA pellet was resuspended in Tris-EDTA buffer (10 mM Tris-HCl, pH 8.0, 1 mM EDTA) supplemented with 0.2 mg/mL RNase A and stored at -20 °C.

### Extraction of plasmid DNA

Plasmid DNA was isolated from recombinant clones of *E. coli* either by alkaline lysis or by using the NucleoBond^®^ Xtra Midi kit from Macherey-Nagel (Düren, Germany) following the manufacturer’s instructions.

### PCR amplification and plasmid constructions

PCRs were conducted using either GoTaq DNA polymerase or Q5^®^ high-fidelity DNA polymerase. Reaction setups, components, and thermocycling conditions followed the guidelines provided by New England BioLabs (Evry, France). All primers, synthesized by IDT, are listed in **Supplementary Table 1**. PCR-amplified fragments and endonuclease-digested products were purified using the Macherey Nagel Gel and PCR Clean-Up Kit, according to the manufacturer’s instructions. The backbone plasmid PV189, previously described (Yaakoub et al., 2025), comprises a hygromycin B resistance cassette (hereafter referred to as Hyg^R^) with the *hph* gene conferring resistance to this drug. The disruption cassette was performed by amplifying 1137 bp of the 5’ UTR and 1272 bp of the 3’ UTR flanking the *sch9* ORF (contig SEQ_SAPIO_0088, positions 2078350–2081181) using the primer pairs SCH9 5’ F/SCH9 5’ R and SCH9 3’ F/SCH9 3’ R, which introduced ClaI/HindIII and NotI/BstXI restriction sites, respectively (**Supplementary Figure 1**). Both UTR fragments were cloned into their respective endonuclease sites in the PV189 plasmid, resulting in plasmid PV189/*Δsch9*, which contains the Hyg^R^ cassette arranged as *5’-sch9-Hyg^R^-3’-sch9* (**Supplementary Figure 1**).

### Fungal transformation

Fungal transformation of *S. apiospermum* was performed on protoplasts using the standard PEG-CaCl_2_ chemical method as described by Liu et al (Liu and Friesen, 2012). For the *sch9* gene disruption experiment, protoplasts of the *Δku70* strain were transformed with 10 μg of the linear *5’-sch9-Hyg^R^-3-sch9* cassette, which was released by digesting PV189 with the ClaI restriction enzyme. Transformants were selected by plating protoplasts on molten agar supplemented with 50 μg/mL hygromycin B. Disruption of *sch9* was validated using PCR and RT-PCR. Standard PCR was run using the primers SCH9_Verif_F1 and SCH9_Verif_R1, which anneal to the *sch9* flanking regions and serve to confirm proper insertion of the disruption cassette. Primers annealing within the *sch9* coding sequence (SCH9_qpcr_F1 and SCH9_qpcr_R1) were used for RT-PCR to verify the absence of *sch9* expression. The aforementioned primers are listed in **Supplementary Table 1**.

### Macroscopic and microscopic analysis

Macroscopic analysis was conducted on 14-day-old colonies grown at 37 °C on YPDA and Malt agar (malt extract, 15 g/L; and chloramphenicol, 0.5 g/L). Microscopic examination was performed using the classical Scotch tape method, with mycelium stained with bromophenol blue.

### Growth and germination kinetics

For growth kinetics studies, colony diameters were measured daily for 14 days following the spotting of 5 μL of a conidial suspension (5.10^5^/mL) onto the center of YPDA plates, which were then incubated at 37 °C. For germination assays, cultures were prepared in YPD broth (yeast extract, 10 g/L; peptone, 20 g/L; glucose, 20 g/L; and chloramphenicol, 0.5 g/L) inoculated with 5.10^5^ spores/mL. Suspensions were then distributed into 12-well plates and incubated under normoxic, hypoxic, or hypercapnic conditions at 37 °C. Germ tube formation was monitored microscopically at 5, 8, 10 and 16 hours post incubation and quantified using ImageJ. We also checked the conidial cell size and granularity using flow cytometry analysis. To do so, a conidial suspension (5.10^5^/mL) was prepared in phosphate-buffered saline (PBS) and analyzed on FACSCanto II cytometer (Becton-Dickinson, Franklin Lakes, NJ, USA), measuring the forward (FSC) and side scatter (SSC) signals.

### Susceptibility to stress conditions

The viability of conidia under various stress conditions was assessed by spotting 5 μL of serial 1:10 dilutions (ranging from 2.10^7^ to 2.10^1^ spores/mL) onto YPDA plates (controls) or onto YPDA plates supplemented with different concentrations of stress-inducing agents: cumene hydroperoxide (0.5 and 1 mM), diamide (0.5 and 1 mM), H_2_O_2_ (0.25 and 0.5 mM), menadione (25 and 50 μM), paraquat (0.5 mM), KCl (25 and 100 mM), NaCl (25 and 100 mM), sorbitol (0.5 M), caffeine (10 mM), Congo red (500 μg/mL), and calcofluor white (70 μg/mL). The plates were incubated at 37 °C for 3 days. All reagents were supplied by Sigma-Aldrich (Saint-Louis, MO, USA).

### Antifungal susceptibility testing

*In vitro* susceptibility testing was conducted following the instructions of both Clinical and Laboratory Standards Institute (CLSI) M38 reference protocol and the European Committee on Antimicrobial Susceptibility Testing (EUCAST) E.Def 9.4 protocol. Freshly prepared conidia were suspended in RPMI-1640 (Sigma-Aldrich), buffered with morpholino-propane sulfonic acid (MOPS) (Sigma-Aldrich) at a final concentration of 0.165 M (pH 7) and supplemented with 0.1% Tween-80. The experiments were carried out in 96-well flat-bottom microdilution plates with a final volume of 200 μL per well. Conidia were tested at a final concentration of 2.10^5^ conidia/mL against drugs at concentrations ranging from 15.625 ng/mL to 16 μg/mL. After incubating the microplates for 72 hours at 37 °C, growth was assessed by spectrophotometric measurement at 405 nm. The MIC50 was defined as the lowest concentration of a drug that inhibited 50% of the growth compared to the drug-free control well.

### Transmission electron microscopy

Conidia recovered from a 9-day-old culture were prepared for transmission electron microscopy (TEM) analysis following the method described by Ghamrawi et al (Ghamrawi et al., 2014). Thin sections were obtained and examined using a JEM-1400 Transmission Electron Microscope (Jeol, Paris, France) operating at 120 kV. Only longitudinal sections were considered for evaluating cell wall thickness.

### Reverse transcription – quantitative PCR

For gene expression quantification, strains were grown on YPDA plates and incubated at 37 °C for 6 to 10 days under normoxic, hypoxic, or hypercapnic conditions. Fungal mycelium was harvested daily, ground in liquid nitrogen, and total RNA was extracted using the Macherey Nagel RNA NucleoSpin RNA Plant and Fungi extraction kit (Düren, Germany). RNA samples (500 ng) were then reverse transcribed (RT) using the SuperScript™ II Reverse Transcriptase kit (Invitrogen, Cergy-Pontoise, France). Gene expression levels were then analyzed using quantitative PCR (qPCR) with a StepOne Plus Thermal Cycler (Applied Biosystems, Foster City, CA, USA). RT-qPCR was performed with 2X Fast SYBR^®^ Green Master Mix (Applied Biosystems) following the manufacturer’s instructions. Genes encoding actin were used for normalization. Relative quantification (RQ) of gene expression was determined from the threshold cycle (Ct) values using the formula described by Livak and Schmittgen (Livak and Schmittgen, 2001). The efficiencies of primers and the stability of the reference gene under the conditions tested for RT-qPCR were validated as previously described (Elhaj Mahmoud et al., 2023).

#### Phagocytosis assay

Cell Preparation and Co-Culture: Peripheral blood mononuclear cells (PBMCs), specifically monocytes, were isolated from healthy human donors and differentiated into M1 phenotype macrophages in complete RPMI 1640 (Lonza, Basel, Switzerland) containing 50 ng/mL granulocyte-macrophage colony-stimulating factor (GM-CSF) as previously described (Paolini et al., 2020). Freshly harvested conidia suspended in PBS were stained with 40 μg/mL fluorescein isothiocyanate (FITC) (Sigma-Aldrich) for 30 min in darkness with constant shaking (120 rpm), washed twice with PBS, and enumerated. FITC-labeled conidia (5.10^5^ cells) were co-cultured with M1 macrophages (1:1 cell ratio) in a 6-well plate with a total volume of 500 μL of complete RPMI-1640. The co-culture was incubated for 6 hours at 37 °C in 5% CO_2_. Following incubation, the plates were placed on ice for 20 minutes to halt phagocytosis and then processed for either ingestion or killing assays. At least two independent replicates with technical triplicates in each were carried out for each strain. Ingestion and killing assays were done as previously described by Staerck et al (Staerck et al., 2021).

Ingestion and killing assays: For the ingestion assays, macrophages were stained with anti-CD14 monoclonal antibody (mAb) (Miltenyi Biotec, Paris, France) and assessed for viability using 7-aminoactinomycin D (7-AAD) (Invitrogen). The ingestion percentage was calculated as the number of live macrophages that had ingested conidia (CD14+ FITC+) divided by the total number of live macrophages (CD14+), multiplied by 100. For the killing assays, intracellular conidia released from phagocytes were labeled with 25 μg/mL propidium iodide (PI) (Sigma-Aldrich). The percentage of killing was calculated as the number of macrophage-killed conidia (FITC+ PI+) divided by the total number of conidia released upon macrophage lysis (FITC+), multiplied by 100. All samples were analyzed using a FACSCanto II cytometer (Becton-Dickinson, Franklin Lakes, NJ, USA).

### Statistical analysis

Statistical tests were performed using GraphPad Prism 8.0. Two-way Anova analysis was primarily used to evaluate differences between the mutant and its parent strain, followed by Bonferroni posttests performed for kinetics studies. TEM data were analyzed using the parametric t-test after checking the normal distribution of the samples. Results were considered statistically different when *p* < 0,05. Unless otherwise stated, all experiments were performed using three independent biological replicates (i.e., independent experiments conducted on different dates using distinct biological samples), each including at least three technical replicates (i.e., repeated measurements of the same biological sample).

## Results

### Identification of a *sch9* homolog gene in *S. apiospermum*

The complete Open Reading Frame (ORF) of *S. apiospermum sch9 (*Sapio_CDS3554, XP_016644332.1) was retrieved from the NCBI database by performing BLAST using *sch9* sequences from *Candida albicans* (*Candida albicans* SC5314, XP_720953.2) and *Aspergillus fumigatus* (*Aspergillus fumigatus* Af293, XP_750389.1) a queries. Protein Blast of Sapio_CDS3554 encoded protein revealed 69% query coverage and 57.7% identity with CaSch9 and 66% query coverage and 62.52% identity with AfSch9. SMART analysis revealed that Sapio_CDS3554 deduced protein sequence harbors the three regions commonly found in Sch9 proteins i.e. (i) a protein kinase C conserved region 2, (ii) a serine/threonine protein kinase catalytic domain, and (iii) an extension to the serine/threonine-type protein kinase.

### Disruption of the *sch9* gene

To investigate the role of Sch9 in *S. apiospermum*, we used the *Δku70* parent strain to create a *sch9* knockout via homologous recombination using a specific disruption cassette (**Supplementary Figure 2A**). After numerous fungal transformation experiments, we were able to isolate a single *Δsch9* clone. PCR screening using primers annealing to the gene flanking extremities was run to confirm the proper knockout of *sch9* and replacement by the Hyg^R^ disruption cassette. The mutant single clone revealed a 9.5 kb PCR fragment at the *sch9* locus, indicating the presence of the disruption cassette. Conversely, the parent strain exhibited a 4.5 kb fragment corresponding to the intact *sch9* gene (**Supplementary Figure 2B**). RT-qPCR also confirmed *sch9* disruption in this clone; while the *sch9* mRNA in the *Δku70* strain was detected by RT-qPCR, expression of *sch9* was completely abolished in the *Δsch9* strain in all of the three tested conditions (normoxia, hypoxia, hypercapnia) (**Supplementary Figures 2C**, **S2E**, **S3D**).

### Macroscopic and microscopic aspects of the *Δsch9*

**Figure 1:**
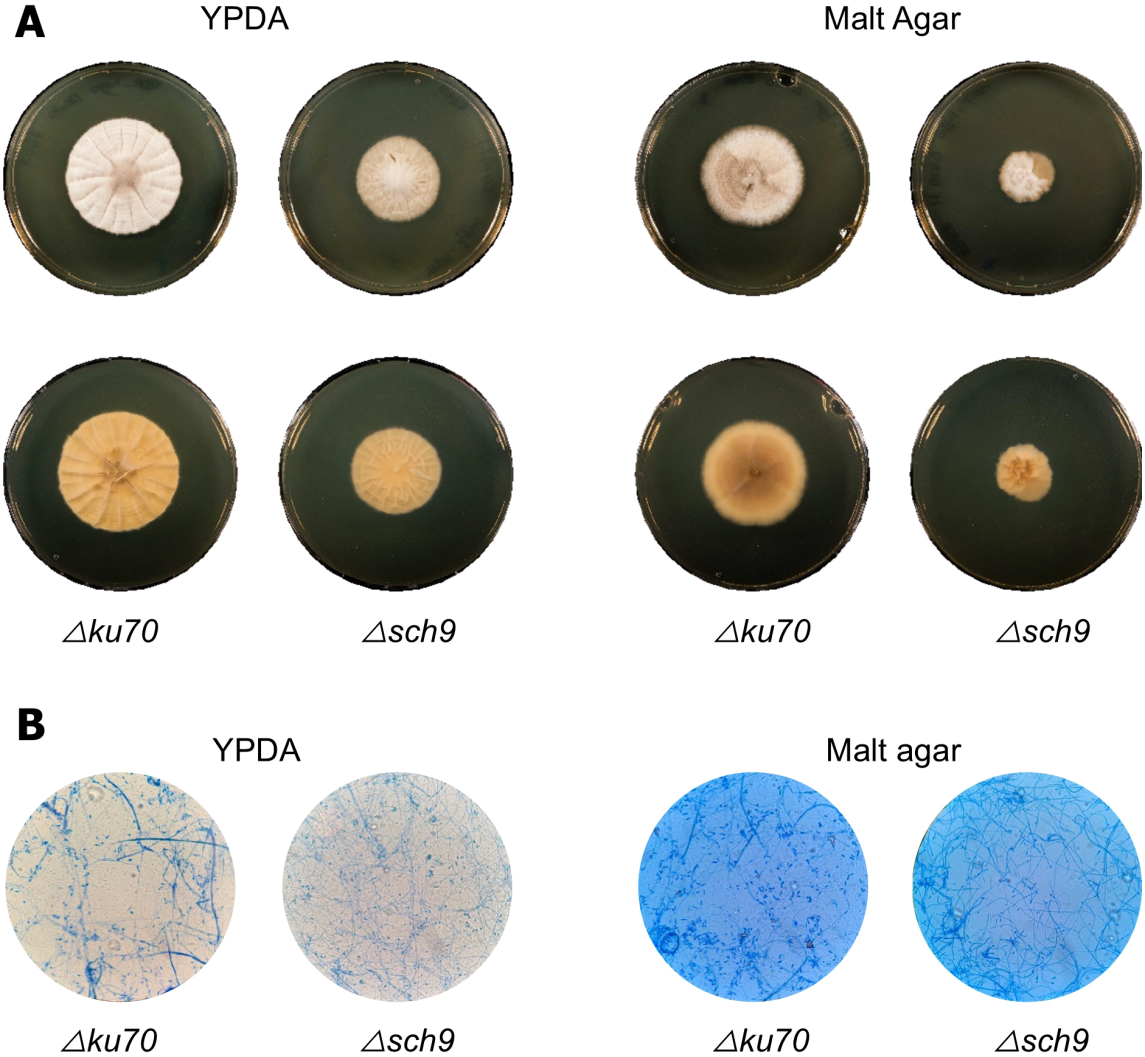
Macroscopic and microscopic appearance of the *Δsch9* mutant and its *Δku70* parent strain. **(A)** Appearance of colonies grown on YPDA and MALT agar after 14 days of incubation at 37 °C (upper pane, surface; lower panel, reverse). **(B)** Microscopic examination of the *Δsch9* mutant and the parent strain following culture on YPDA or Malt agar for 14 days at 37 °C. Examination was performed using the classical Scotch tape method with bromophenol blue staining.

To check whether Sch9 plays a role in *S. apiospermum* development, macroscopic and microscopic features of the fungus were assessed. On YPDA and Malt agar, *Δsch9* colony exhibited smaller and less cottony colonies than *Δku70* (**Figure 1A**). Interestingly, differences in colony pigmentation were also observed between *Δsch9* and the parent strain. Apart from *Δsch9* displaying thinner filaments, no major differences between the two strains were observed at the microscopic level (**Figure 1B**). These results suggest that Sch9 regulates growth in *S. apiospermum*, potentially aerial development.

### Cell structure and architecture

**Figure 2:**
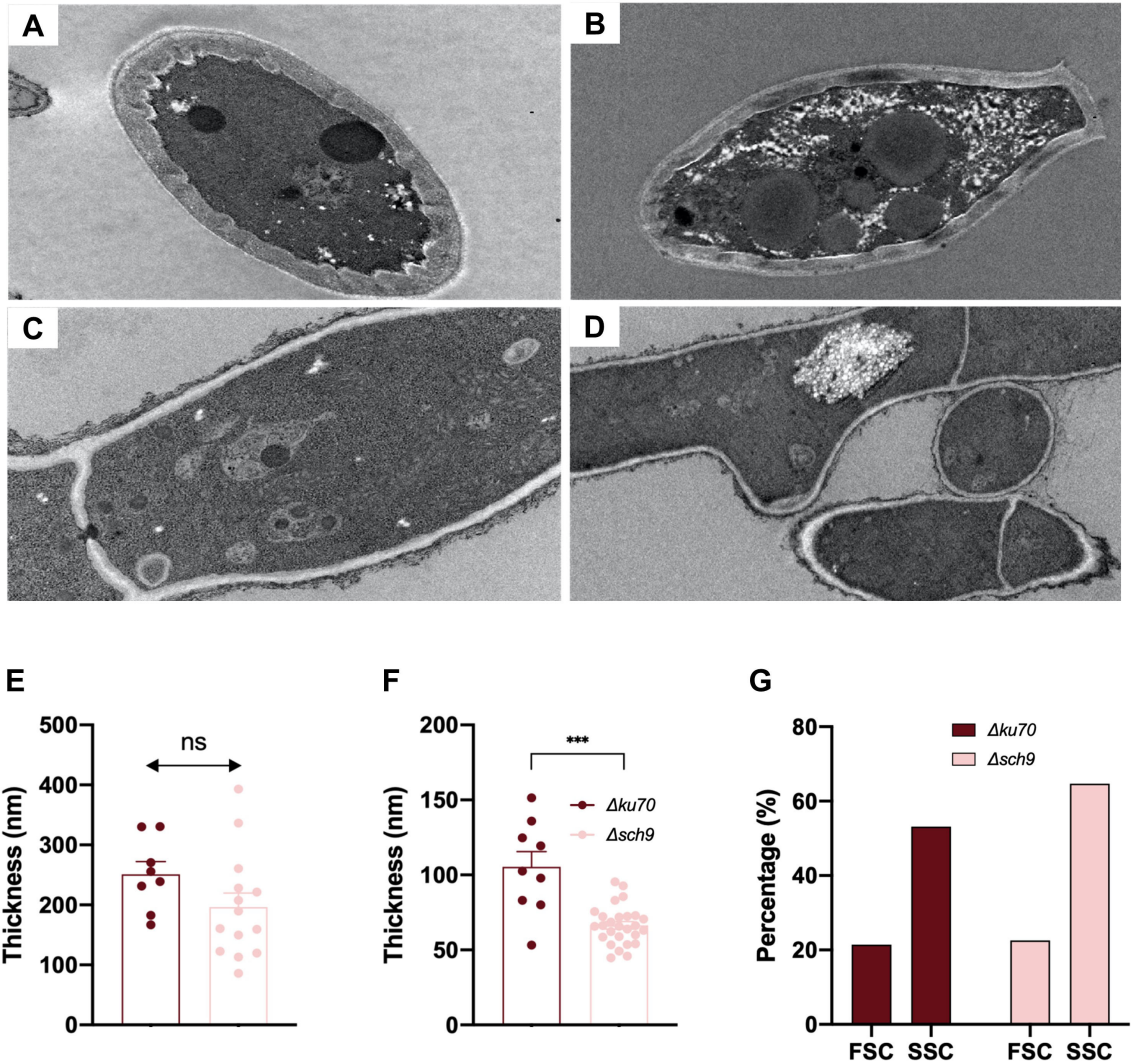
Conidia structure and architecture of the *Δsch9* mutant and its *Δku70* parent strain. TEM observation of spores **(A, B)** and germ tubes **(C, D)** of the mutant and the parent strain. Conidia recovered from a 9-day-old culture were prepared for transmission electron microscopy (TEM) analysis following the method described by Ghamrawi et al (Ghamrawi et al., 2014). Thin sections were obtained and examined using a JEM-1400 TEM operating at 120 kV. The thickness of the conidial **(E)** and hyphal **(F)** cell wall in *Δsch9* and *Δku70*. Statistical analyses were performed using parametric t-tests due to the normal distribution of samples. The data presented in the graphs are derived from measurements taken on individual cells when ultrastructural features allowed accurate determination of cell wall thickness. Only longitudinal sections were considered for evaluating cell wall thickness. **(G)** Flow cytometry analysis showing forward scatter (FSC) and side scatter (SSC) signals in *Δsch9* and *Δku70* conidia, indicative of relative differences in cell size and granularity. * P ≤ 0.05, ** P ≤ 0.01, *** P ≤ 0.001.

TEM examination (**Figures 2A–D**) showed that conidia and germ tubes exhibited ultrastructure features previously reported for *Scedosporium boydii* (Ghamrawi et al., 2015), including homogeneous cytoplasm, and regularly shaped mitochondria and nuclei. However, most *Δsch9* conidia displayed a thinner outer electron-dense cell wall layer compared to the parent strain. Notably, both conidia and germ tubes of the *Δsch9* mutant showed an accumulation of white reticular organelles of unknown nature and function, which were not observed in the parent strain.

TEM analysis also revealed differences in cell wall thickness between the *Δsch9* mutant and the parent strain (**Figures 2E, F**). Interestingly, *Δsch9* germ tubes showed significantly thinner cell wall (66.37 nm ± 12.86 nm) compared to the parent strain (105.4 nm ± 30.73 nm). On the other hand, the cell wall of *Δsch9* conidia was slightly thinner than that of *Δku70*, however, this difference was not statistically significant. These findings infer that Sch9 plays a role in hyphal cell wall synthesis.

### Conidia size and granularity

TEM did not provide sufficient data to assess differences in conidial size. Therefore, to further investigate potential variations in conidial size and granularity, flow cytometry was employed for a more comprehensive analysis (**Figure 2G**). Flow cytometry analysis revealed that the forward scatter (FSC) intensity, which reflects cell diameter, was similar between the *Δsch9* and the *Δku70* parent strain. However, the *Δsch9* exhibited an increased side scatter (SSC) intensity compared to *Δku70*, indicating greater internal granularity. These findings are consistent with the TEM findings, further supporting the observed differences in cellular architecture.

### Growth analysis

**Figure 3:**
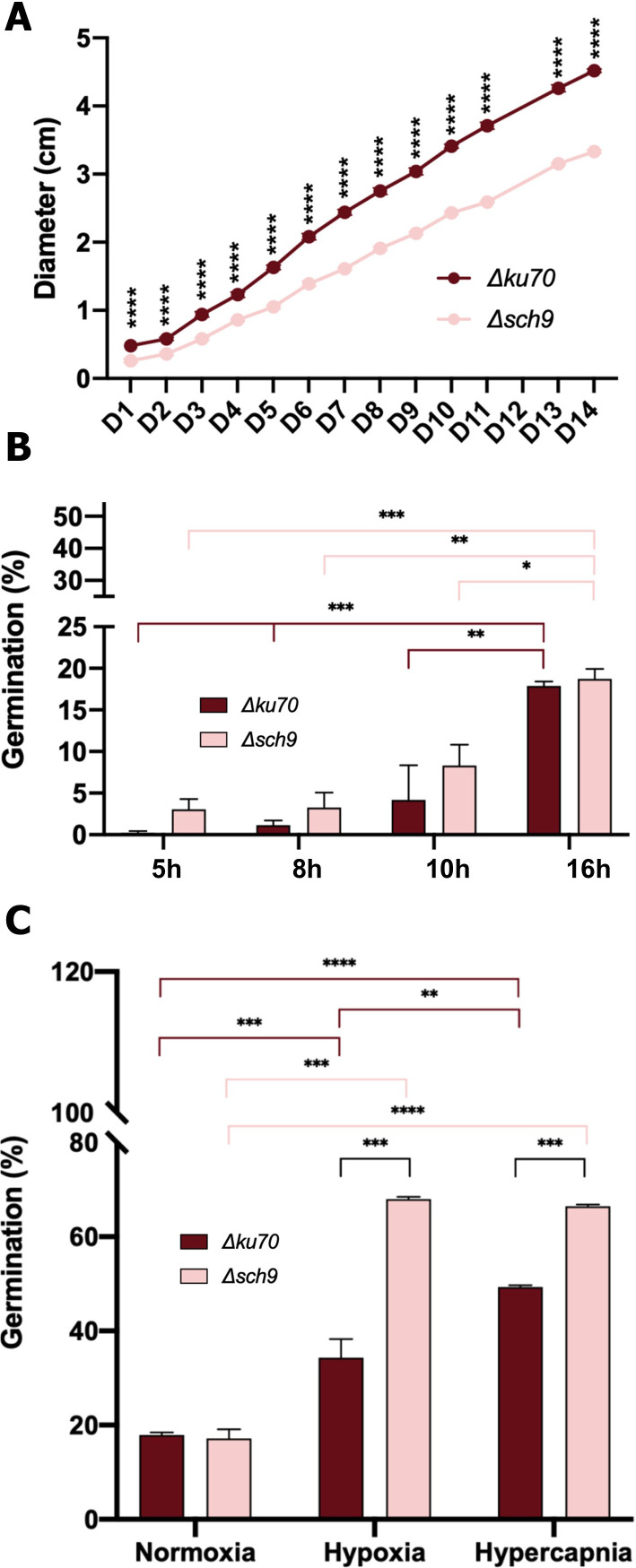
Growth and germination kinetic rates of the *Δsch9* mutant strain and its *Δku70* parent strain. **(A)** Growth kinetics of the mutant and its parent strain represented by colony diameter. Colony diameters were measured daily for 14 days after spotting 5 μL of a conidial suspension (5.10^5^/mL) onto the center of YPDA plates incubated at 37 °C. Five technical replicates were conducted to acquire these results. **(B)** Germination kinetics of the mutant and its parent strain. Liquid cultures were performed in YPD broth and incubated at 37 °C, and germination was monitored microscopically at 5, 8, 10 and 16 hours of incubation. **(C)** Germination rate (16h) measured under different gaseous conditions. Cultures were performed in YPD broth and incubated for 16 h at 37 °C under normoxia, hypoxia or hypercapnia. Germination was checked following microscopic examination. Statistical analyses were performed using Graphpad prism 8.0 two-way ANOVA tests. * P ≤ 0.05, ** P ≤ 0.01, *** P ≤ 0.001.

Given that reduced mycelial growth was observed in the *Δsch9* strain compared to the parent strain grown on YPDA and Malt agar (**Figure 1**), growth kinetics were analyzed to further investigate the effect of *sch9* deletion on the growth patterns (**Figure 3A**). Monitoring growth on YPDA revealed a significant reduction in colony size of *Δsch9* compared to the *Δku70* at all time points. The difference in growth was already evident at early time points and progressively increased throughout the time. All together, these results suggest that Sch9 contributes to the overall fitness in *S. apiospermum*.

### Germination analysis

Given the difference in growth kinetics observed at early time points, germination kinetics were subsequently assessed. When cultured in YPD broth at 37 °C, both *Δsch9* and *Δku70* strains exhibited a progressive and similar increase in germination rates, as shown in **Figure 3B**.

Given the key role that Sch9 plays in gas sensing in fungi, germination of *Δsch9* and the parent strain was also evaluated under these conditions. After 16h of incubation, the germination rate of *Δsch9* was significantly higher under hypoxia (67.9% ± 0.870%) and hypercapnia (66.4% ± 0.613%) compared to normoxia (17.3% ± 3.352%) (**Figure 3C**). Although the *Δku70* parent strain also showed enhanced germination under hypoxia and hypercapnia compared to normoxia, this effect was significantly amplified in the *Δsch9* mutant (34.3% vs. 67.9% for hypoxia and 49.3% *vs*. 67.9% for hypercapnia, respectively). These results indicate that Sch9 contributes to the regulation of germination in *S. apiospermum* under hypoxic or hypercapnic but not under normoxic conditions.

### Susceptibility to various stress conditions

Despite the inherent fitness defect of the *Δsch9* affecting its strain growth in our drop-plate assay, the susceptibility of *Δsch9* to NaCl, KCl, and sorbitol was intact, suggesting that Sch9 does not participate in the adaptation of *S. apiospermum* to hyperosmotic conditions (**Supplementary Figure 3**).

**Figure 4:**
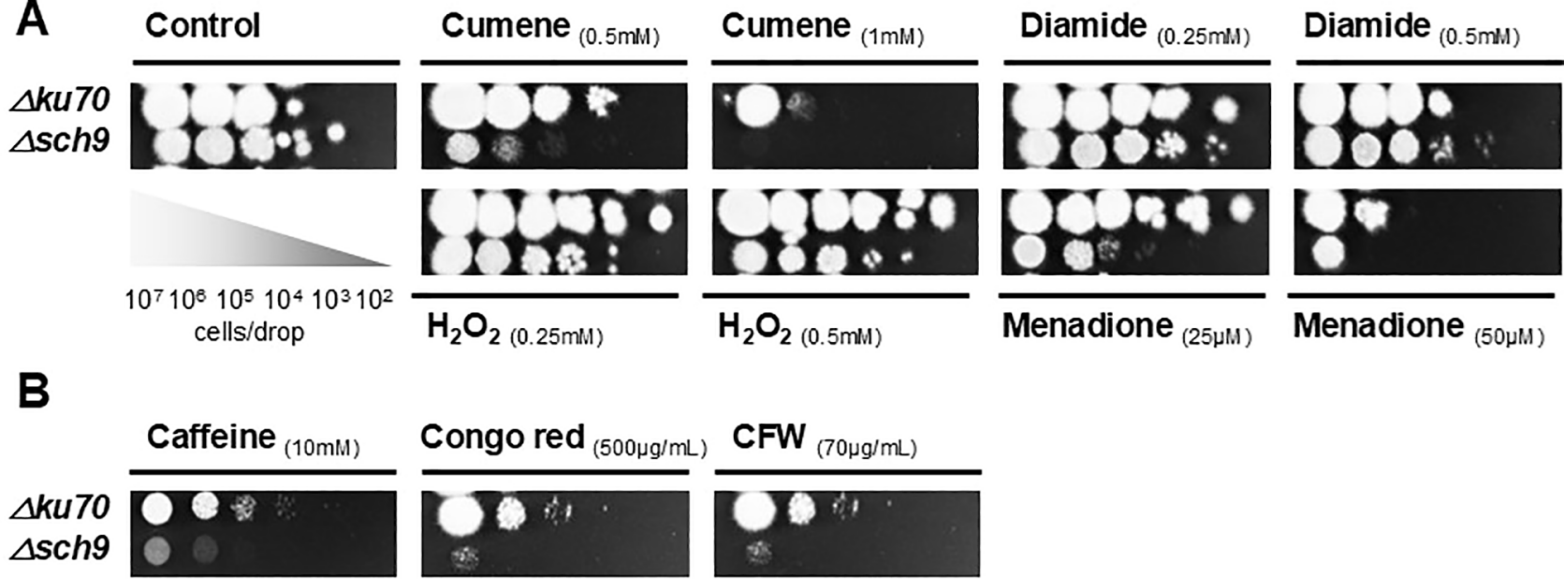
Susceptibility to various stress conditions of the *Δsch9* mutant and its *Δku70* parent strain. **(A)** Susceptibility testing to oxidative stress. **(B)** Susceptibility testing to cell wall stress. The viability of the conidia was analyzed by spotting 5 μL of serial 1:10 conidial dilutions (from 2.10^7^ to 2.10^1^ spores/mL) on YPDA plates supplemented with different concentrations of stress-inducing reagents, or on stress-free YPDA plates used as a control. Plates were incubated at 37 °C for 3 days.

The role of *sch9* in resistance to oxidative stress was evaluated using various agents, including cumene hydroperoxide, menadione, H_2_O_2_, and diamide (**Figure 4A**). We detected an increased sensitivity of the *Δsch9* strain to cumene hydroperoxide and menadione compared to the parent strain. These findings indicate that *sch9* protects *S. apiospermum* from oxidative stress imposed by certain agents.

The effect of cell wall stress-inducing agents was also assessed. Congo red and calcofluor white, which bind strongly to the cell wall polysaccharides and therefore disrupt the cell wall, were tested, along with caffeine, which specifically targets the TOR signaling pathway by inhibiting TORC1. The *Δsch9* exhibited increased sensitivity to each of these cell wall-perturbing agents (**Figure 4B**). These findings suggest that the deletion of *sch9* led to a potential defect in cell wall integrity which resulted in increased sensibility to cell wall agents., It is important to note that the heightened susceptibility to stress agents may be compounded by the overall reduced fitness of the *Δsch9*. Consistent with our TEM observations reporting a thinning of the outer cell wall layer in *Δsch9*, these results highlight a potential role of Sch9 in modulation of *S. apiospermum* cell wall.

### Susceptibility to antifungals

**Figure 5:**
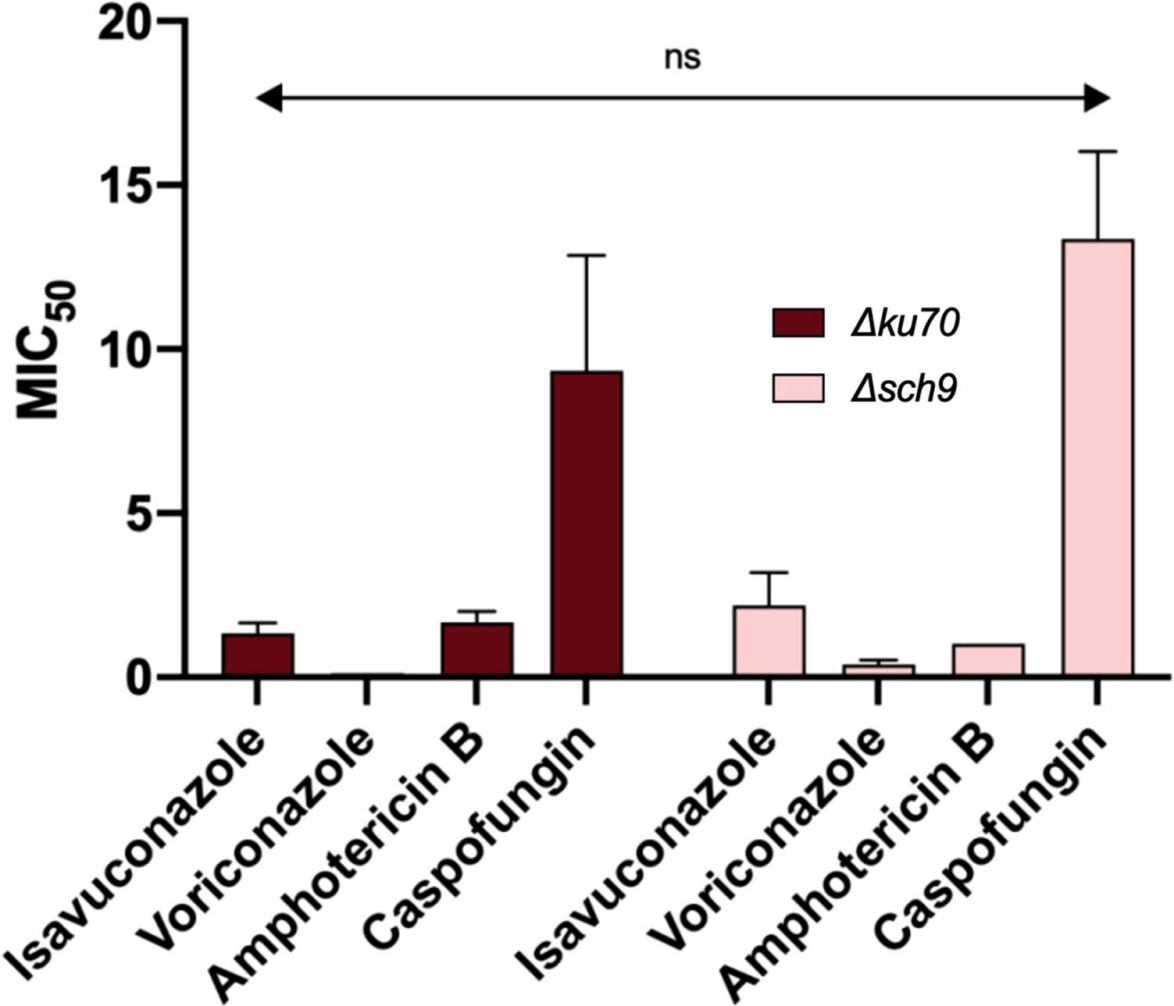
Antifungal susceptibility testing of the *Δsch9* mutant and its *Δku70* parent strain. Susceptibility of strains to antifungals was evaluated using the microdilution antifungal susceptibility testing following CLSI and EUCAST recommendations with minor modifications. Statistical analyses were performed using Graphpad prism 8.0 two-way ANOVA tests. *** P ≤ 0.001.

*S. apiospermum* is known to be resistant to most of the current antifungals. The role of *sch9* in regulating tolerance to antifungal drugs was therefore investigated. Susceptibility of the *Δsch9* strain to voriconazole, isavuconazole, amphotericin B, and caspofungin revealed no significant differences compared to the parent strain, indicating that Sch9 does not mediate *S. apiospermum* tolerance to the tested antifungals (**Figure 5**).

### Interaction of *S. apiospermum* with innate immune cells

**Figure 6:**
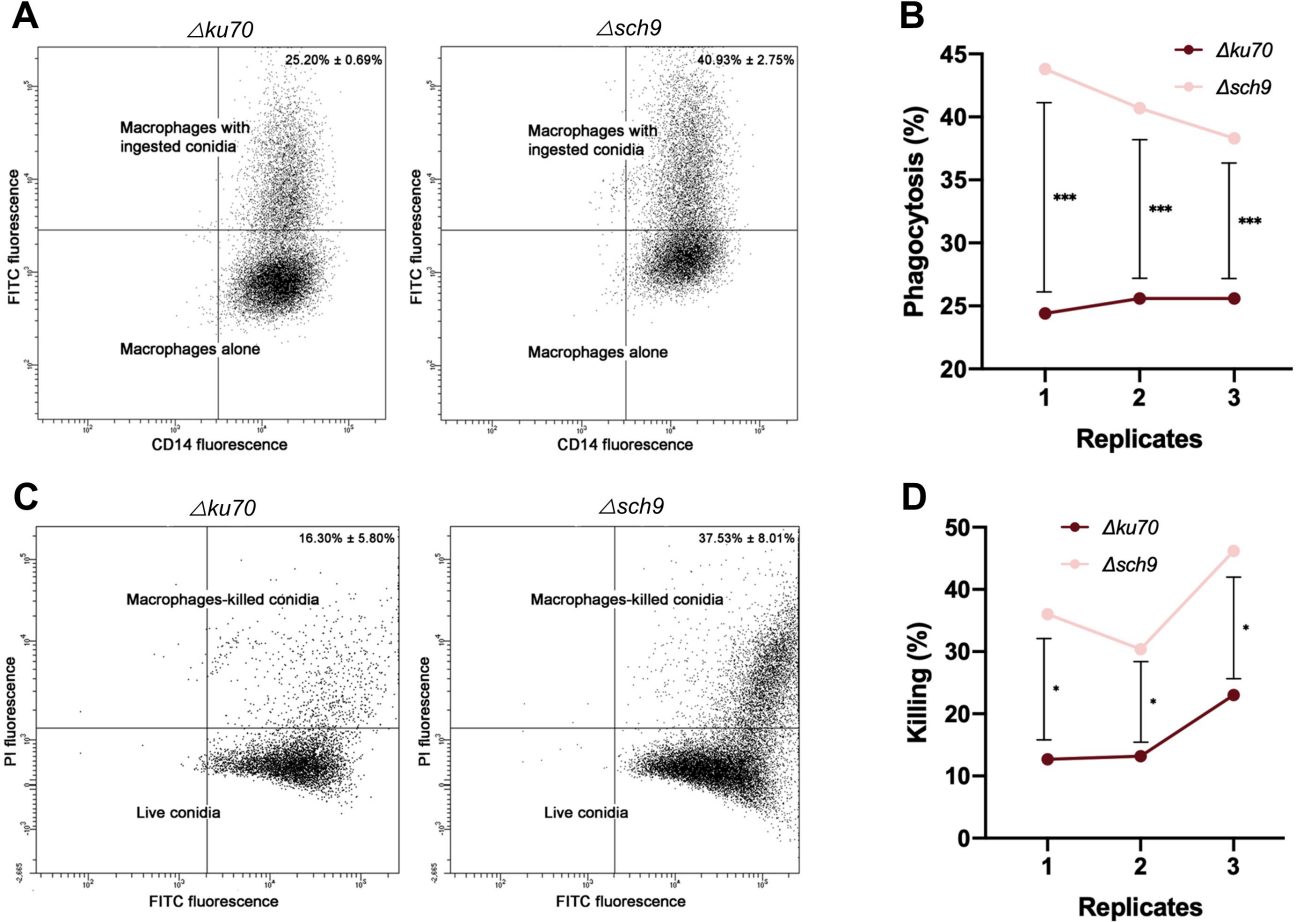
Interaction of the *Δsch9* mutant and its *Δku70* parent strain with innate immune cells. **(A)** Phagocytosis assay: Plots showing conidia-free macrophages (CD14+) and macrophages with ingested conidia (CD14+ FITC+). **(B)** The phagocytosis percentage was calculated as the number of live macrophages that had ingested conidia (CD14+ FITC+) divided by the total number of live macrophages (CD14+), multiplied by 100. **(C)** Killing assay: Plots showing conidia that had been ingested by macrophages and whether survived its attack (FITC+) or got killed (FITC+ PI+). **(D)** The percentage of killing was calculated as the number of macrophage-killed conidia (FITC+ PI+) divided by the total number of conidia released upon macrophage lysis (FITC+), multiplied by 100. Two independent replicates with three technical replicates were performed in each assay. Statistical analyses were performed using Graphpad prism 8.0 two-way ANOVA tests.

The critical role of early germination of *A. fumigatus* spores in their recognition by the host immune system is well-established (Briard et al., 2020). In light of this, we sought to investigate the effect of Sch9 on the interaction between *S. apiospermum* conidia and macrophages. Specifically, we assessed two key parameters: the ingestion of conidia and their subsequent killing by PBMC-derived M1 macrophages. Notably, the ingestion rate of *Δsch9* conidia was significantly higher compared to *Δku70* (40.93 ± 2.75% vs. 25.20 ± 0.69%, respectively) (**Figures 6A, B**).

In addition, the percentage of killing was found more pronounced for *Δsch9* conidia compared to the parent strain (37.53 ± 8.01% vs. 16.30 ± 5.80%, respectively) (**Figures 6C, D**). These results suggest that Sch9 may play a role in protecting the fungus against the host immune response, including phagocytosis and subsequent killing by macrophages. However, given the complexity of immune evasion mechanisms, the increased phagocytosis and killing of *Δsch9* conidia may also reflect altered surface properties rather than a specific evasion mechanism.

## Discussion

The pathogenesis of microorganisms hinges on several critical processes, including (*i*) fungal fitness, defined as the ability of a microorganism to survive and grow within a specific environment and (*ii*) disruption of homeostasis and expression of virulence traits that damage the host (Brunke et al., 2016; Panepinto and Williamson, 2006). In such perspectives, *S. apiospermum* represents an excellent model as an opportunistic filamentous fungus at the origin of respiratory or disseminated infections following inhalation of its conidia in immunocompromised patients, such as those with solid organ transplantation or hematological malignancies. Moreover, *S. apiospermum* is the second most common filamentous fungus capable of colonizing the CF lungs. Thereby, this filamentous fungus has likely developed various molecular mechanisms to evade the host immune response and have established apparent insensitivity to most current antifungal drugs. As a consequence, although some progress has been made with using combinatorial approaches to treat *Scedosporium* infections, the discovery of new therapeutic targets in this opportunistic mold is urgently needed (Bouchara and Papon, 2019; Ramirez-Garcia et al., 2018).

The TOR signaling pathway has been well-established as a crucial regulator of various stress-related and pathogenicity-associated cellular processes in certain opportunistic fungal species (Wang et al., 2023). Sch9 is a major substrate of TOR pathway and has been linked to environment adaptation and virulence in fungi, as well as in CO_2_ sensing (Caligaris et al., 2023; Martin et al., 2017). In this context, the present study aimed to investigate the role of the *sch9* gene in *S. apiospermum*. This pivotal kinase may indeed play a significant role in the adaptation of this fungus to the unique microenvironment encountered in the CF airways. Furthermore, Sch9 could represent a potential therapeutic target for the development of treatments for life-threatening fungal infections.

*S. apiospermum Δsch9* displayed a reduced radial growth that likely derives from a global default in fungal fitness. Similarly, the deletion of the *sch9* gene led to cell growth alteration in *S. cerevisiae*, *F. graminearum*, and *Magnaporthe oryzae* (Pascual-Ahuir and Proft, 2007; Chen et al., 2014; Batool et al., 2022). While the decrease in conidia size was previously shown for the *Δsch9* in *S. cerevisiae*, *C. albicans*, and *F. graminearum*, we did not observe in our experiments any differences in conidia size between the *Δsch9* and the parent strain. This may reflect species-specific functions of Sch9. In particular, it is possible that in *S. apiospermum*, Sch9 contributes to the regulation of cell cycle-associated processes, such as centromere activity during replication, as previously reported in *C. albicans* (Varshney et al., 2015), thereby impacting hyphal extension and radial growth rather than conidial size. Nevertheless, flow cytometry revealed increased SSC values corresponding to increased granularity and complexity in the ultrastructure of the *Δsch9* mutant. These results are in accordance with the occurrence of white reticular organelles in TEM in the *Δsch9*. The latter has not been previously defined, and no function has been assessed to them, thus, their role remains unknown.

Previous studies have shed light on the ultrastructure of dormant and germinating conidia in another *Scedosporium* species, *S. boydii* (Ghamrawi et al., 2014). Fungal cell walls of filamentous Ascomycota are commonly constituted of two superimposed layers, an outer electron-dense protein-rich layer, and an inner electron transparent layer rich in polysaccharides. In *A. fumigatus*, the role of cell wall proteins varies from maintaining cell shape to protecting the cell against foreign substances, mediating adhesion for cell migration and fusion, and transmitting external stimuli to the intracellular environment. They also can act as mediators in absorbing molecules (Linder et al., 2005; Gastebois et al., 2009).

In this study, we pointed out a reduced thickness of the outer cell wall layer in the *Δsch9*. Additionally, the *Δsch9* displayed increased sensitivity to Congo red, Calcofluor white, and caffeine, all of which are known to induce cell wall stress. The role of TOR signaling was found to be essential for ribosome biogenesis and protein synthesis in various eukaryotic models (Wang et al., 2023). Thus, we may hypothesize that the deletion of the *sch9* gene may indirectly impair synthesis of the outer cell wall proteins by disrupting the global translation machinery. This alteration in protein synthesis may be associated with the accumulation of white reticular organelles in the mutant cells. Overall, these cellular dysregulations may contribute to the reduced fitness observed upon *sch9* deletion.

The functional analysis of the *S. apiospermum Δsch9* revealed increased susceptibility to specific stress-inducing agents compared to the parent strain. Notably, while the mutant did not exhibit differential sensitivity to osmotic stress agents, it demonstrated heightened sensitivity to cumene hydroperoxide and menadione, both of which generate reactive oxygen species (ROS) leading to oxidative stress. These results suggest that the *sch9* gene is essential for general stress responses in *S. apiospermum*. However, further experiments are necessary to elucidate the specific role of Sch9 in regulating the stress response pathways in this fungal species. However, further studies are required to elucidate the specific mechanisms by which Sch9 regulates stress response pathways in this species. In particular, it would be of interest to investigate whether Sch9 functionally interacts with or is rewired to MAPK signaling pathways under stress conditions, as reported in other fungal species (Wang et al., 2004; Alves de Castro et al., 2016; Li et al., 2024). Additionally, given the involvement of Sch9 in the regulation of secondary metabolism in fungi (Gu et al., 2015; Li et al., 2024), it would be relevant to determine whether altered production of stress-protective metabolites contributes to the observed phenotypes.

*Scedosporium* species are well described as resistant to amphotericin B and most triazole antifungals, including the most recent triazole drug, isavuconazole. They also display reduced susceptibility to the most recent family of antifungals, i.e., echinocandins, and more particularly to caspofungin. In this regard, voriconazole is recommended, in combination or not with another class of antifungal, by the European guidelines as first-line treatment of *Scedosporium* deep-seated infections (Ramirez-Garcia et al., 2018). Therefore, the treatment of deadly scedosporiosis still relies on few therapeutic options, and the development of therapeutic alternatives is urgently needed. We thus investigated the susceptibility of the *Δsch9* to the four previously mentioned antifungals. Unfortunately, we did not find any differential susceptibility to any of the tested drugs. The Sch9 seems not to participate in the regulation of antifungal susceptibility in *S. apiospermum*. Taken together, these findings suggest that Sch9 is unlikely to represent a suitable target for adjunctive therapies aimed at enhancing the efficacy of current antifungal treatments.

Surprisingly, while the growth rate of the *Δsch9* was reduced at 37 °C, we observed an enhanced germination ability only under hypoxic or hypercapnic conditions. As previously described in *C. albicans*, Sch9 negatively regulates Nce103, a carbonic anhydrase responsible for the conversion of CO_2_ to HCO_3_^-^. CO_2_ has been broadly recognized as a stimulus of morphogenesis in pathogenic fungi (Stichternoth et al., 2011). This aligns with our finding of the enhanced germination observed in the *Δsch9* mutant in *S. apiospermum*. Additional experiments are needed to further elucidate the roles of the various components and their interactions within the TOR signaling pathway in regulating early stress-induced morphogenetic processes in *S. apiospermum*. In addition, in *C. albicans*, Sch9 has been shown to function downstream of sphingolipid-activated Pkh1/2 kinases, integrating membrane lipid status with respect to growth under CO_2_ conditions, stress response, and morphogenesis (Pohlers et al., 2017). It would be interesting to investigate the potential conservation of such cell signaling similarity between yeast and filamentous fungi in future. Such an investigation could be particularly relevant in *Scedosporium*, since this mold displays a remarkable capacity to adapt to hypoxia and hypercapnia, which are hallmarks of the CF bronchial mucus (Bouchara and Papon, 2019).

The conidium is well recognized as the quiescent and primary infective form in filamentous fungi. In *A. fumigatus*, germination of conidia is a crucial step that triggers the activation of innate immunity by stimulating pro-inflammatory signaling pathways (Latgé et al., 2017; Luther et al., 2007). The swelling of the conidia that precedes germ tube emission, is associated to the release of the outer cell wall layer, and therefore to the loss of the immuno-protective melanin and hydrophobins (rodlets in *A. fumigatus*) (Brakhage et al., 2010), leading to an increased exposure of the pathogen associated molecular patterns (PAMPs) such as cell wall β-glucans and galactosaminogalactans (GAGs). Subsequently, phagocytic cells are recruited and recognize PAMPs of the foreign conidia through their pattern recognition receptors (PRRs). Hence, the innate immune system is in turn stimulated and responds by releasing cytokines and interferons. These immune mediators lead to an inflammatory response and activate the adaptive immunity, finally triggering antigen-specific immune responses leading to the killing of the infecting microorganism. A similar mechanism may operate in *S. apiospermum*, which could explain the increased macrophage ingestion and killing of *Δsch9* conidia, potentially linked to the enhanced germination rate observed in this mutant. Otherwise, recent studies have demonstrated that the Sch9-Rca1-Nce103 signaling module significantly influences β-1,3-glucan exposure in response to hypoxia and lactate (Avelar et al., 2024). Thus, the increased phagocytosis observed in the *Δsch9* mutant may result from impaired cell wall masking, leading to enhanced exposure of immunogenic components such as β-1,3-glucan and, consequently, increased recognition by macrophages. Moreover, the increased killing may also result from the higher sensitivity of the *Δsch9* mutant to oxidative stress, thereby reducing its ability to withstand macrophage-mediated killing.

In the airways of patients with CF, defect in the chloride channel leads to important physicochemical changes in the bronchial mucus. In order to maintain osmotic equilibrium, the defective efflux of anions results in the mucus becoming dehydrated, which profoundly disturbs gas exchange in this sticky mucus. Measurement of the oxygen level within the mucopurulent secretions revealed a 70-fold reduction in oxygen partial pressure compared to the airway lumen, together with a significant increase in carbon dioxide levels (Worlitzsch et al., 2002). To chronically colonize the airways, microorganisms must be able to adapt to this specific microenvironment. Previous transcriptomic experiments revealed that cultivating the fungus in such physicochemical conditions resulted in the downregulation of a large number of genes that are potentially involved in pathogenesis. This includes several genes that are involved in sphingolipid or ergosterol synthesis, the glycosylphosphatidylinositol (GPI)-anchor protein biosynthetic pathway, and secondary metabolism (Vandeputte et al., 2020; Carvalho et al., 2026). These biochemical changes have been shown to markedly impact the virulence of other fungal pathogens, for example by affecting their adherence to epithelial cells or evasion of the host immune response. The present results suggest that adaptation to hypoxia and hypercapnia may be mediated by the Sch9 signaling pathway, since disruption of the corresponding gene led to an increased germination rate under hypoxic or hypercapnic conditions, as well as increased susceptibility to chemically induced oxidative stress and cell wall stress agents. This was accompanied by alterations in cell wall synthesis and hyphal elongation. By regulating hyphal growth and possibly the synthesis of membrane lipids, GPI-anchored proteins, or secondary metabolites with immunomodulatory properties, Sch9 could enable the fungus to persist in the respiratory tract. This could explain the “saprophytic” growth of the fungus which is usually well tolerated in the phases of pulmonary stability of the ecological Climax-Attack model.

The main limitation of this study is that it is based on comparisons only between the *Δku70* parental strain and a single *Δsch9* mutant strain. Ideally, a reintegrant strain would provide definitive proof that the observed phenotypes are specifically attributable to the *sch9* gene deletion. However, *Scedosporium* is well documented to display broad resistance to xenobiotics, particularly antifungals. After testing around ten selection drugs commonly used in fungal genetics for fungal transgenesis experiments, we found that *Scedosporium* is only susceptible to hygromycin B and, to a lesser extent, phleomycin. Our overall strategy, which we initiated over ten years ago with the aim of developing the first genetic transformation technique for *Scedosporium*, was first based on using knockout cassettes that exploit the hygromycin B resistance gene in a wild-type (WT) strain. However, our preliminary experiments clearly showed that transformation rates were extremely low and ectopic recombination was major, leaving little chance of obtaining any targeted mutant. This bottleneck led us to select a single strain harboring a deletion for the *ku70* gene (which is involved in non-homologous DNA break repair) using the phleomycin resistance marker. This *Δku70* recipient strain lacks NHEJ mechanisms and is therefore unable to ectopically integrate inactivation cassettes for a target gene based on the hygromycin B resistance gene. However, the transformation rates of the *Δku70* strain are very low and a vast number of genetic transformation experiments on *Scedosporium* protoplasts (*Scedosporium* displays very low sporulation-conidiation) must be undertaken to ultimately select a single hygromycin-resistant clone that possesses a knockout locus of the targeted gene. In the present study, the phleomycin resistance marker was used to select the *Δku70* parental strain, while the hygromycin B resistance marker was used to generate the *Δsch9* mutant. As a result, no additional selectable markers were available to enable functional reintegration of a wild-type copy of the *sch9* gene into the *Δsch9* mutant.

However, (i) our PCR-based molecular validation clearly shows a mutated *sch9* locus; (ii) the expression levels of the *sch9* transcript in the *Δsch9* strain are undetectable under many culture conditions; (iii) some of the stress susceptibility phenotypes (e.g. oxidant sensitivity) which are observed in the *Scedosporium Δsch9* strain have also been observed in the corresponding mutant in other fungal models; (iv) other stresses, such as hyperosmotic conditions which trigger distinct well-known cellular signaling pathways (e.g. Sho1- and Sln1-HOG), have no differential effect on the *Δku70* and *Δsch9* strains. Therefore, the *Scedosporium Δsch9* mutant strain is not chronically sensitive to all stresses, but is selectively susceptible to stresses that have, in some cases, been observed in *Δsch9* strains of other yeasts and filamentous species. Therefore, it is highly likely that all the observed phenotypes in this mutant strain are indeed linked to the deletion of the *sch9* gene in *Scedosporium*. Previous work by Varshney et al. (2015) in *C. albicans* has shown that loss of *sch9* can lead to chromosome instability and loss. This raises the possibility that some of the observed phenotypes in the corresponding *Scedosporium* mutant could be influenced by underlying genomic instability rather than by defects in signaling pathways. However, we never observed such consistent phenotypes or variation in the *Δsch9* mutant which might lead us to believe that it could be attributed to genomic instability.

In summary, the deletion of *sch9* significantly impaired fitness under standard growth conditions, primarily resulting in reduced hyphal development. Additionally, the *Δsch9* exhibited increased sensitivity to various oxidative and cell wall stress-inducing agents. The deletion of *sch9* enhanced germ tube formation particularly under hypoxic and hypercapnic conditions. Moreover, under standard growth conditions, the *Δsch9* displayed reduced germ tube cell wall thickness compared to the parent strain. Finally, *sch9* disruption compromised the ability of *S. apiospermum* to resist ingestion and killing by macrophages during co-culture. These data suggest that Sch9 may play a critical role in stress tolerance, morphogenesis, and host-pathogen interactions in *S. apiospermum*. Further studies are warranted to explore the potential of targeting *sch9* for therapeutic interventions against *S. apiospermum* infections.

## Data Availability

The original contributions presented in the study are included in the article/**Supplementary Material**. Further inquiries can be directed to the corresponding author.
