# Supplementary material for: Deciphering the role of the Sch9 serine/threonine kinase in Scedosporium apiospermum
Source: Front Fungal Biol. 2026 May 11;7:1820509. doi: 10.3389/ffunb.2026.1820509 (PMC13199340; doi:10.3389/ffunb.2026.1820509)
Supplement: Supplementary file 1 [file DataSheet1.docx]

*Supplementary tables and figures*

**Deciphering the role of the Sch9 serine/threonine kinase in *Scedosporium apiospermum***

Samar Kabbara^1,†^, Hajar Yaakoub^1^, Baptiste Bidon^1,§^, Charlotte Godon^1^, Julia Razafimandimby^1^, Yves Delneste^2^, Pascale Pignon^2^, Jean-Philippe Bouchara^1^, Monzer Hamze^3^, Nicolas Papon^1,^*

_____________________________________________________________________________

**Affiliations**

^1^ Univ Angers, Univ Brest, IRF, SFR ICAT, 49000, Angers, France.

^2^ Univ Angers, Nantes Université, Inserm, CNRS, CRCI2NA, SFR ICAT, F-49000 Angers, France.

^3^ Laboratoire Microbiologie Santé et Environnement (LMSE), Ecole Doctorale des Sciences et de Technologie, Faculté de Santé Publique, Université Libanaise, Tripoli, Lebanon.

*Correspondence: [nicolas.papon@univ-angers.fr](mailto:nicolas.papon@univ-angers.fr)

† Current address: Univ Sorbonne Paris Nord - U1349 Inserm, Bobigny, France.

§ Current address: Centre for Genomics and Precision Medicine, National Taiwan University, Taipei, Taiwan

**_____________________________________________________________________________**

Keywords: Sch9, TOR pathway, *S. apiospermum*, functional characterization, fungal fitness.

**Supplementary Table S1: List of primers.**

| Primer | Sequence from 5’ to 3’ |
| --- | --- |
| SCH9 5' F | GCGCAAAATCGATgacaagtatcttgaatgctctggg |
| SCH9 5' R | GCGCAAAAAGCTTgtaatctcttgtcgtgtaagtccg |
| SCH9 3' F | GCGCAAAGCGGCCGCggctgactctcctttcatcgtcgg |
| SCH9 3' R | GCGCAAACCACCGCGGTGGagtgacgaagaggctgtgtggtca |
| SCH9_Verif_F1 | tgtctagaaatctggtcttcgtcc |
| SCH9_Verif_R1 | caggggttaacctcactcaagccg |
| SCH9_qpcr_F1 | tcgacatgtccgaggaacac |
| SCH9_qpcr_R1 | ccgccttcaggaatcggaat |


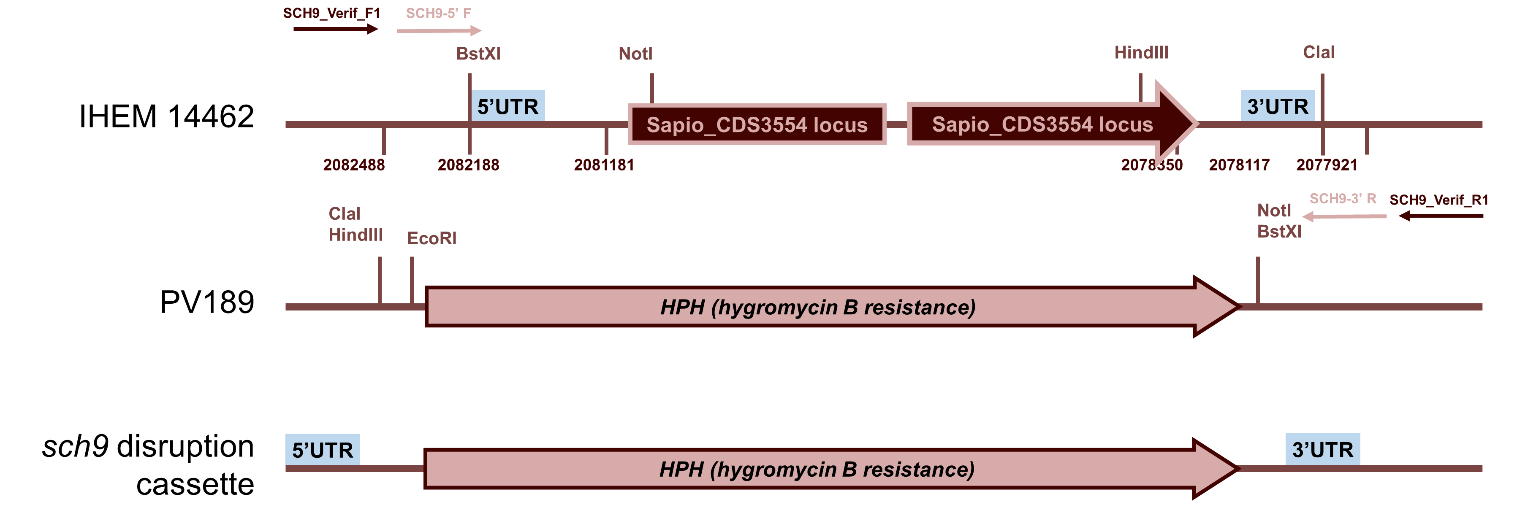


**Supplementary Figure S1: Representation of the *sch9* gene locus, the PV189 backbone plasmid, and the disruption cassette for the *sch9* gene.** The disruption cassette was created by amplifying 1137 bp of the 5' UTR and 1272 bp of the 3' UTR of the *sch9* ORF flanking regions (contig SEQ_SAPIO_0088, positions 2078350–2081181) using the primer pairs SCH9 5' F/SCH9 5' R and SCH9 3' F/SCH9 3' R, which introduced ClaI/HindIII and NotI/BstXI sites, respectively. The backbone plasmid PV189 comprises a hygromycin B resistance cassette (Hyg^R^) with the *HPH* gene conferring resistance to this drug. Both UTR fragments were cloned into their respective endonuclease sites in the PV189 plasmid, giving rise to the plasmid PV189/*Δsch9*, containing the Hyg^R^ cassette, i.e. *5’-sch9-Hyg^R^-3’-sch9.*


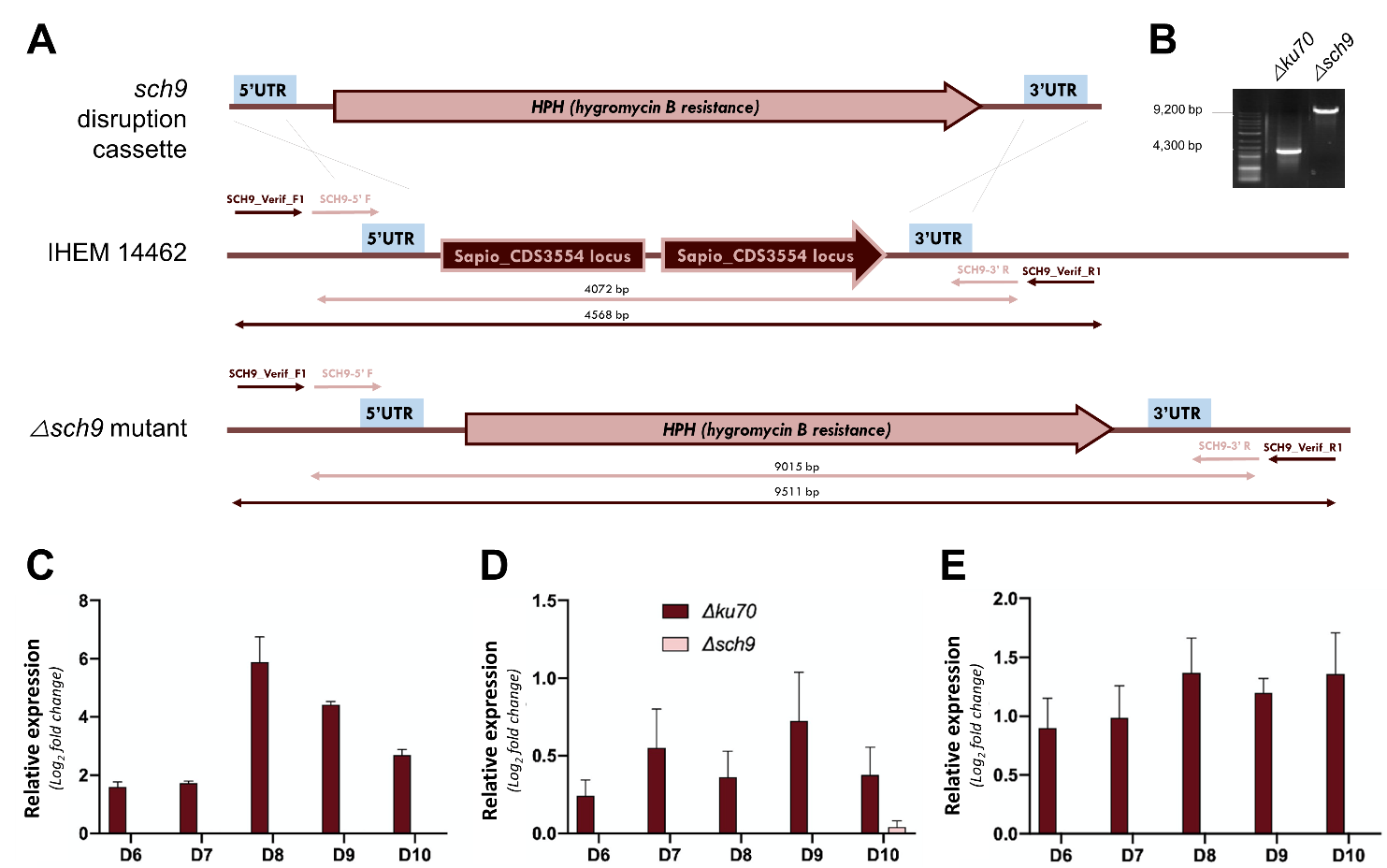


**Supplementary Figure S2:** **Molecular characterization of the *Δsch9* mutant.** (A) Schematic representation of the wild-type and disrupted *sch9* locus. (B) PCR analysis of the *sch9* locus in the parent strain (*Δku70*) and the *Δsch9* by using SCH9_Verif_F1/SCH9_Verif_R1 primers situated before and after the 5’ and 3’ flanking regions respectively. The verification was done by studying the relative expression of the *sch9* gene by RT-qPCR compared to the parent strain. Kinetics (day 6 to day 10) of the relative expression was studied in three different conditions. Results correspond to the relative expression of the *sch9* gene in the mutants and the parent strain under normoxia (C), hypoxia (D) or hypercapnia (E).


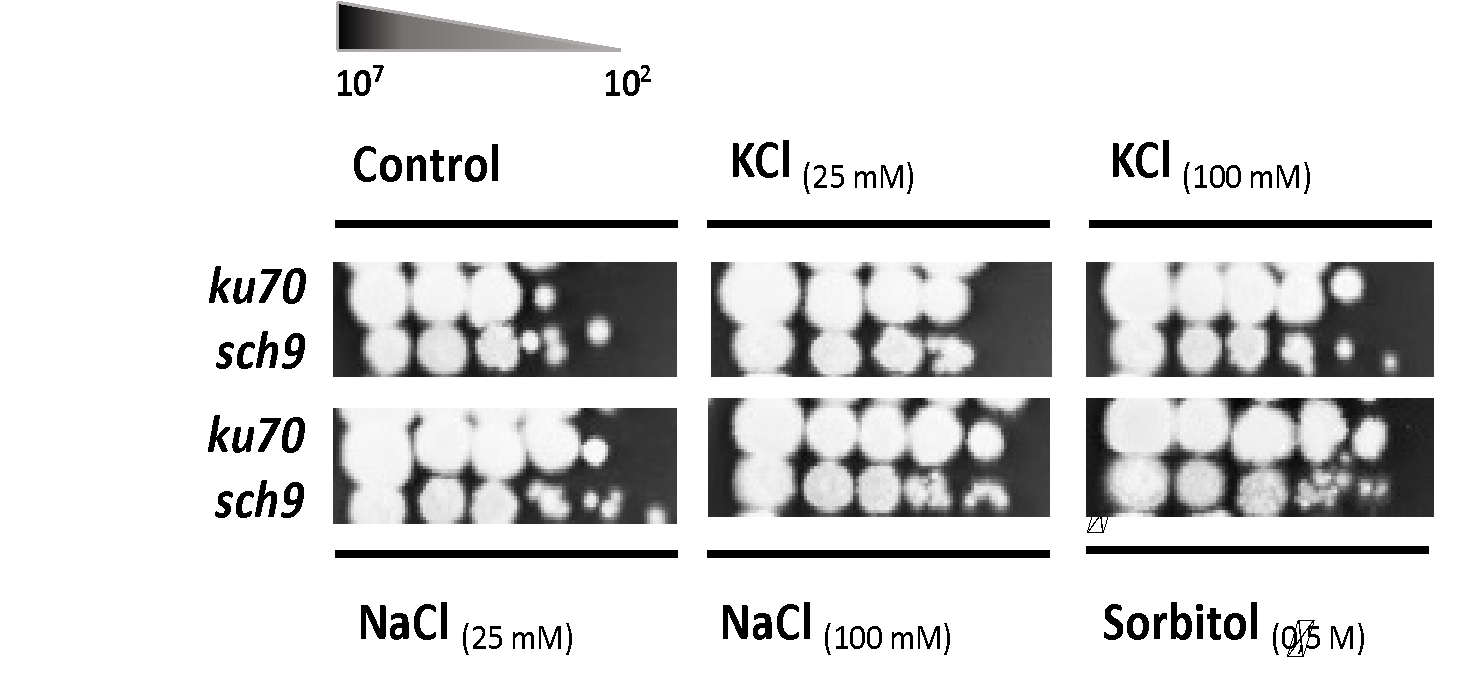


**Supplementary Figure S3:** **Susceptibility testing to osmotic stress.** The viability of the conidia was analyzed in different stress conditions by spotting 5 μL of serial 1:10 conidial dilutions (from 2.10^7^ to 2.10^1^ spores/mL) on YPDA plates as a control, or on YPDA plates supplemented with different concentrations of stress causing reagents. Plates were incubated at 37°C for 3 days.
